# Transcriptional Changes Caused by Bisphenol A in *Oryzias javanicus*, a Fish Species Highly Adaptable to Environmental Salinity

**DOI:** 10.3390/md12020983

**Published:** 2014-02-14

**Authors:** Seonock Woo, Vianney Denis, Seungshic Yum

**Affiliations:** 1 South Sea Environment Research Division, Korea Institute of Ocean Science and Technology, Geoje 656-830, Korea; E-Mail: cwoo@kiost.ac; 2 Biodiversity Research Centre, Academia Sinica, Taipei 115, Taiwan; E-Mail: vianney.denis@gmail.com

**Keywords:** *Oryzias javanicus*, cDNA microarray, bisphenol A, gene expression, salinity

## Abstract

The Javanese medaka, *Oryzias* *javanicus*, is a fish highly adaptable to various environmental salinities. Here, we investigated the effects of the environmental pollutant bisphenol A (BPA; an endocrine disrupting chemical) on gene expression levels in this species acclimated to different salinities. Using cDNA microarrays, we detected the induction of differential expression of genes by BPA, and compared the transcriptional changes caused by chemical exposure at different salinities. There were marked transcriptional changes induced by BPA between treatments. While 533 genes were induced by a factor of more than two when *O. javanicus* was exposed to BPA in seawater, only 215 genes were induced in freshwater. Among those genes, only 78 were shared and changed significantly their expression in both seawater and freshwater. Those genes were mainly involved in cellular processes and signaling pathway. We then categorized by functional group genes specifically induced by BPA exposure in seawater or freshwater. Gene expression changes were further confirmed in *O. javanicus* exposed to various concentrations of BPA, using quantitative real-time reverse transcription PCR based on primer sets for 28 selected genes.

## 1. Introduction

Global concern about endocrine disrupting chemicals (EDCs) has been increasing because of their potential to cause hormonal imbalance in natural systems. Exposure to environmental estrogen is associated with abnormal physiological changes and reproductive impairment in many animals [1,2,3]. Some studies have suggested that chemical toxicity tends to be enhanced by salinity, and have indicated that biotransformation together with other dispositional factors may be important in this phenomenon [4,5,6,7]. To investigate the effects of an EDC on gene expression levels in an organism acclimated to different salinities, we used microarray experiments to compare the expression of genes induced in *Oryzias javanicus* (a fish species highly adaptable to environmental salinity) following exposure to bisphenol A (BPA) in seawater (SW) and freshwater (FW).

The genus *Oryzias* comprises more than 14 species (including the well-known model laboratory fish *O. latipes*), and each species occurs specifically in SW, brackish water, or FW [8]. Because of their distribution patterns in various salinities, it has been suggested that *Oryzias* species are useful for studies of diversity, osmotic regulation/adaptation, ecology, and evolution. Amongst the genus *Oryzias*, the Javanese (or marine) medaka *O. javanicus*, and *O. dancena* are highly adaptable to environmental salinity [9]. However, in both SW and FW, *O. javanicus* has higher fertilization and hatching rates than *O. latipes* or *O. dancena*, and a lower death rate. In addition, *O. javanicus* has many advantages as a model marine fish, including its small size, its transparent eggs and body, and its stability in laboratory aquaculture.

In the marine environment, toxic chemicals including EDCs are generally found at relatively low concentrations because of dilution effects in the ocean. Amongst EDCs, BPA is an industrial chemical used in production of epoxy resins and polycarbonate plastics. BPA has been found in the environment, and has been the subject of considerable research into its potential effects on aquatic organisms, including its capacity to influence reproduction, hatching, morphogenesis, and mortality [10]. As BPA tends to bind to estrogen receptors and shows prolonged activity, there is the potential for production of biologically active intermediates, which if formed at particular developmental stages, can lead to secondary physiological imbalances and a wide range of adverse effects, including impaired brain function and disturbance of the immune system [11].

The objectives of this study were: (i) to investigate the effects of BPA on transcription in *O. javanicus*; (ii) to facilitate identification of biomarker genes for detecting BPA contamination in the marine environment; and (iii) using *O. javanicus* acclimated in FW, to seek unique transcription signatures under diverse salinity conditions for assessing the environmental risks of a toxic chemical originating on land but transported to the ocean.

## 2. Results and Discussion

Coastal and estuarine environments commonly fluctuate with respect to salinity. Consequently, animals used for environmental risk assessment in such environments must be highly adaptable to salinity if their laboratory responses to pollutants are to be comparable to those under field conditions. The relationships among the observed accumulation of EDCs in fish, the environmental concentrations of EDCs, and the biological effects of EDC exposure have been investigated in many organisms, including our recent report of expression profiling in Javanese medaka exposed to 17β-estradiol [12,13,14]. However, the influence of salinity on the expression of genes in organisms exposed to EDCs has only recently been investigated [15,16]. Here, we investigated the effect of salinity on BPA toxicity using the Javanese medaka, a fish highly adaptable to environmental salinity. The present study is the first to report marked transcriptional changes induced by BPA under different salinity conditions, using cDNA microarrays.

The number of differentially expressed genes (DEGs) induced in Javanese medaka exposed to BPA greatly differed between the SW and FW treatments (Table 1). Of the 533 genes induced in SW, 289 genes were upregulated and 244 were downregulated by a factor of more than two in *O. javanicus* exposed to BPA. In freshwater, only 215 genes were induced with 101 genes being upregulated and 114 downregulated. Approximately 23% of DEGs from the fish exposed in SW and 27% from the fish exposed in FW were not well characterized, and were clustered as a general functional prediction only, or were classified as “unknown”. Respectively for the fish exposed to BPA in SW and FW, 34% and 31% among the well characterized DEGs were related to cellular process and signaling, and 39% and 31% were associated with transduction mechanisms. A total of 78 DEGs were common to fish exposed to BPA in the SW and FW treatments, 455 were specific to those exposed in SW, and 137 were specific to those exposed in FW. Most of the DEGs in fish exposed in SW were related to signal transduction mechanisms, translation, ribosomal structure/biogenesis, and posttranslational modification/chaperones; these comprised >32% of the total DEGs. A large number of DEGs (>25% of the total) found in fish exposed to BPA in FW were related to signal transduction mechanisms, lipid transport and metabolism, translation, and ribosomal structure/biogenesis.

A total of 39 DEGs changed by a factor of more than three in fish exposed to BPA in either or both SW and FW. This concerned 28 genes in fish exposed to BPA in SW, and 11 genes in fish exposed to BPA in FW (Supplementary Table S1). There was a significant difference between the expression of 13 of these genes in SW and FW: chymotrypsinogen 2-like protein, uridine phosphorylase 2, TBT-binding protein, ATP citrate lyase, heat shock protein 84b, decorin, telomerase reverse transcriptase, glycerol-3-phosphate dehydrogenase, glutathione S transferase Rho-class, apolipoprotein A-IV4, and 2 other unknown genes.

For example, TBT-binding protein mRNA was upregulated by a factor of approximately four in fish exposed to BPA in SW, while in fish exposed to FW, the increase was a factor of approximately two. However, for other DEGs showing an increase by a factor of more than three in fish exposed to BPA in SW, no significant change was found in fish exposed in FW, and *vice versa*. For example, choline kinase alpha mRNA was upregulated by a factor of approximately three in fish exposed to BPA in FW, but no significant change in expression of this gene was found in fish exposed to BPA in SW. A review of studies of the influence of salinity on the toxicity of various chemicals to a variety of organisms [17] showed that negative correlations (toxicity increasing with decreasing salinity) are most frequently reported (55%), followed by no correlation (27%) and positive correlations (18%). Amongst these studies, many showed that for most metals, toxicity increased with decreasing salinity, but there was generally no consistent trend for the toxicity of most organic chemicals with changing salinity [18]. Because there are few transcriptomic studies with which to compare our results, it may be premature to reach conclusions about the effects of salinity on enhancement or inhibition of the toxicity of BPA in *O. javanicus*. However, our microarray data clearly suggest that more major gene expression changes were induced in fish exposed to BPA in SW than in FW.

**Table 1:** Functional clustering of differentially expressed genes by BPA in *O. javanicus* in seawater and freshwater (FC > 2).

COG Groups *	Description	SW		FW	
		Gene Count	(%)	Gene Count	(%)
Information storage and processing	Translation, ribosomal structure and biogenesis	56	15	16	11
	RNA processing and modification	11		2	
	Transcription	8		4	
	Replication, recombination and repair	3		0	
	Chromatin structure and dynamics	1		1	
Cellular processes and signaling	Cell cycle control, cell division, chromosome partitioning	14	34	3	31
	Defense mechanisms	19		11	
	Signal transduction mechanisms	69		21	
	Cell wall/membrane/envelope biogenesis	2		0	
	Cell motility	0		1	
	Cytoskeleton	14		10	
	Extracellular structures	3		1	
	Intracellular trafficking, secretion, and vesicular transport	12		6	
	Posttranslational modification, protein turnover, chaperones	46		14	
Metabolism	Energy production and conversion	35	28	15	31
	Carbohydrate transport and metabolism	11		7	
	Amino acid transport and metabolism	25		9	
	Nucleotide transport and metabolism	7		2	
	Coenzyme transport and metabolism	0		1	
	Lipid transport and metabolism	38		17	
	Inorganic ion transport and metabolism	9		6	
	Secondary metabolites biosynthesis, transport and catabolism	22		9	
Poorly characterized	General function prediction only	20	23	17	27
	Function unknown	108		42	
Total	-	533	100	215	100

FC, fold change. * Clusters of Orthologous Groups of proteins.

Secondly, a difference related to salinity was found in the functional clustering of DEGs in fish exposed to BPA in the SW and FW treatments. Approximately 32% of DEGs in fish exposed to BPA in SW were related to signal transduction mechanisms, translation/ribosomal structure, and posttranslational modification/protein turnover/chaperone functions. However, approximately 32% of DEGs in fish exposed to BPA in FW were related to lipid transport/metabolism and energy production/conversion, as well as signal transduction mechanisms and translation/ribosomal structure. In addition, the number of genes involved in defense mechanisms, and energy production and conversion was relatively high in fish exposed to BPA in FW, and a few genes including those coding for BRCA1 protein, nuclear receptor interacting protein 2, and synovial sarcoma translocation protein were only found in fish exposed to BPA in SW. BRCA1 is known to have a role as a tumor suppressor (producing DNA repair protein) and as a co-regulator in the transcriptional response to DNA damage [19,20]. We found that expression of the BRCA1 gene was repressed by a factor of approximately two in *O. javanicus* exposed to BPA in SW, relative to the non-exposed control group, but was not changed in fish exposed to BPA in FW. Another tumor-related gene, encoding synovial sarcoma translocation protein, was repressed only in *O. javanicus* exposed to BPA in SW. The synovial sarcoma translocation protein belongs to a transcriptionally active nuclear complex, and it was recently reported that its depletion in primary synovial sarcoma cells contributes to tumor development in part through beta-catenin signaling [21]. The major focus of research on the toxic effects of BPA has been on its endocrine disrupting activity, but the findings reported here of a positive correlation between salinity and BPA toxicity suggest that BPA exposure under high salinity conditions might be associated with repression of tumor suppressing genes, and associated tumorigenesis in marine organisms.

A third difference in the effect of salinity on *O. javanicus* exposed to BPA was the common occurrence of DEGs in fish exposed in the SW and FW treatments. Most of the DEGs common to each BPA exposure group were related to signal transduction mechanisms and metabolism. Among the 78 common DEGs, 34 were similarly upregulated or downregulated in fish exposed to BPA in SW or FW (Table 2). These genes usually showed similar expression levels in fish exposed in both SW and FW, exceptions were the genes encoding decorin, TBT-binding protein, and an unknown gene similar to *Tetraodon nigroviridis* full-length cDNA. While the expression of the two later genes for fish in SW was approximately twice as high as in FW, the decorin gene was upregulated by a factor of more than two in FW (relative to fishes exposed to BPA in SW). Decorin is a small proteoglycan that has been found to be associated with collagen fibrils in all connective tissues, and it also interacts with the complement component C1q, epidermal growth factor (EGF), and transforming growth factor-beta (TGF-beta) [22,23]. In addition, there are reports that decorin may be involved in regulation of the cell cycle because it enhances or inhibits TGF-beta 1 activity [24,25,26]. The finding of upregulation of decorin genes in fish exposed to BPA in either SW or FW in this study implies that exposure to a low concentration of BPA is sufficient to alter the transcriptional response, which could affect cell proliferation and the risk of cancer.

**Table 2:** The differentially expressed genes by BPA exposure in both SW and FW with the same expression tendency (FC > 1.5).

Functions	Best Hit Description	SW	FW
Information storage and processing	*Crocodylus porosus* 28S ribosomal RNA	+1.84 ± 0.07	+1.71 ± 0.03
	*Oncorhynchus mykiss* 28S ribosomal RNA	+1.82 ± 0.05	+1.83 ± 0.04
	*Solea senegalensis* ribosomal protein S25	−1.84 ± 0.07	−1.72 ± 0.07
Cellular processes and signaling	*Danio rerio* fibronectin 1b	−1.84 ± 0.07	−2.05 ± 0.03
	*Danio rerio* nardilysin	−1.91 ± 0.08	−1.62 ± 0.03
	*Danio rerio* signal recognition particle 72	−1.63 ± 0.01	−1.52 ± 0.04
	*Oncorhynchus mykiss* leukocyte elastase inhibitor	−2.03 ± 0.04	−1.63 ± 0.05
	*Oreochromis niloticus* decorin	+2.82 ± 0.07	+6.74 ± 0.04
	*Oryzias javanicus* complement C5 precursor	+1.51 ± 0.09	+2.05 ± 0.03
	*Oryzias javanicus* peptidylprolyl isomerase domain and WD repeat containing 1	−1.55 ± 0.06	−1.54 ± 0.02
	*Paralichthys olivaceus* complement component C8 beta	+2.01 ± 0.04	+1.52 ± 0.01
	*Paralichthys olivaceus* fibrinogen beta chain precursor	+2.22 ± 0.07	+1.52 ± 0.07
	*Salmo salar* homocysteine-responsive endoplasmic reticulum-resident ubiquitin-like domain member 1 protein	−1.51 ± 0.04	−1.55 ± 0.06
	*Salmo salar* transforming growth factor-beta-induced protein ig-h3	+2.02 ± 0.03	+1.54 ± 0.07
Metabolism	*Anoplopoma fimbria* ATP synthase lipid-binding protein, mitochondrial precursor	+1.55 ± 0.05	+1.56 ± 0.08
	*Chaetodon mertensii* cytochrome P450 CYP2N	+1.61 ± 0.01	+1.54 ± 0.07
	*Danio rerio* 5,10-methylenetetrahydrofolate reductase-like	−2.04 ± 0.06	−2.33 ± 0.04
	*Homo sapiens* ATP citrate lyase, transcript variant 2	+2.82 ± 0.04	+1.52 ± 0.07
	*Oryzias javanicus* angiotensin I converting enzyme (peptidyl-dipeptidase A) 1	+1.51 ± 0.05	+2.01 ± 0.07
	*Oryzias javanicus* arylamine *N*-acetyl transferase	−1.82 ± 0.02	−2.11 ± 0.08
	*Osmerus mordax* glycerol-3-phosphate dehydrogenase, cytoplasmic	−3.04 ± 0.03	−1.84 ± 0.04
	*Paralichthys olivaceus* fibrinogen beta chain precursor	+2.21 ± 0.07	+1.54 ± 0.07
	*Paralichthys olivaceus* ornithine decarboxylase antizyme large isoform	−1.91 ± 0.05	−1.53 ± 0.08
	*Takifugu rubripes* apolipoprotein A-IV4	+2.92 ± 0.04	+3.92 ± 0.09
Poorly characterized	*Anoplopoma fimbria* nuclear protein 1	−2.94 ± 0.01	−1.52 ± 0.05
	*Fundulus heteroclitus* TBT-binding protein	+4.21 ± 0.04	+1.81 ± 0.04
	*Homo sapiens* genomic sequence surrounding NotI site, clone NL6-EJ23R	+1.62 ± 0.01	+1.54 ± 0.08
	*Oryzias javanicus* BCSC-1 isoform 1	−1.83 ± 0.08	−1.74 ± 0.02
	*Oryzias javanicus* choriogenin H	−1.82 ± 0.01	−2.04 ± 0.03
	*Oryzias javanicus* tumor suppressor	−1.51 ± 0.05	−1.54 ± 0.03
	*Tetraodon nigroviridis* full-length cDNA	−1.71 ± 0.02	−2.04 ± 0.05
	*Tetraodon nigroviridis* full-length cDNA	−1.62 ± 0.03	−1.63 ± 0.04
	*Tetraodon nigroviridis* full-length cDNA	+3.82 ± 0.08	+1.54 ± 0.01

SW, seawater; FW, freshwater; +, upregulation; −, downregulation.

However, some of the common DEGs showed opposite trends in expression in fish exposed to BPA in the SW and FW treatments (44 DEGs, Table 3). Approximately 60% of those genes characterized cellular processing and signaling group, and many were related to defense mechanisms. For example, the alpha-2-macroglobulin, complement C3-S, and complement factor H-related protein genes were upregulated by a factor of more than two in fish exposed to BPA in SW, but were downregulated by a factor of more than two in fish exposed in FW. Especially, the expression of the heat shock protein 84b and glutathione S transferase (GST) genes increased by a factor of more than five in fish exposed to BPA in SW (+5.51 ± 0.03 and +6.94 ± 0.06, respectively), but only by a factor of approximately two in fish exposed maintained in FW. The upregulation of expression of the GST gene in *O. javanicus* has previously been reported in response to exposure to organophosphorus pesticides and heavy metals, where its transcription increased in specific tissues in a dose-dependent manner [27,28]. The response of the GST gene in oysters exposed to diesel oil was increased by increased salinity, suggesting that salinity influenced the diesel-related biomarker responses and toxicity [29]. Transcriptional changes in heat shock proteins have also been reported in several studies of Javanese medaka exposed to cadmium, benzo(a)pyrene, and iprobenfos, and heat shock protein genes were reported to be upregulated or downregulated by exposure to specific chemicals [30,31]. The delta 6-desaturase gene was also upregulated by a factor of approximately two in fish exposed to BPA in SW relative to the control fish, but downregulated by a factor of more than six in fish exposed in FW, relative to non-exposed control fish. In contrast, the telomerase reverse transcriptase gene was strongly repressed in fish exposed to BPA in SW, but induced in fish exposed in FW. Reductions or enhancement of telomerase activity or telomerase reverse transcriptase gene expression have been reported in stress-related studies including pollutant exposure, ageing, and disease, and it has been suggested that changes in activity or expression levels could be used as stress-specific indicators [32,33,34].

**Table 3:** The differentially expressed genes by BPA exposure in both SW and FW with opposite expression tendency (FC > 1.5).

Functions	Best Hit Description	SW	FW
Information storage and processing	*Oryzias javanicus* 40S ribosomal protein S18	−2.41 ± 0.03	+1.81 ± 0.08
	*Oryzias javanicus* 60S ribosomal protein L23	−1.92 ± 0.07	+1.82 ± 0.04
	*Pimephales promelas* BTEB transcription factor	−1.93 ± 0.01	+1.81 ± 0.01
	*Salmo salar* ribosomal protein L13a	−1.82 ± 0.05	+1.82 ± 0.06
	*Scophthalmus maximus* 60S ribosomal protein L14	−1.84 ± 0.09	+1.92 ± 0.03
	*Solea senegalensis* ribosomal protein L13	−2.01 ± 0.09	+1.83 ± 0.09
Cellular processes and signaling	*Carassius auratus* protein phosphatase 2 regulatory subunit B beta	−2.52 ± 0.08	+1.84 ± 0.07
	*Danio rerio* RAB8A, member RAS oncogene family	−1.83 ± 0.07	+1.83 ± 0.01
	*Danio rerio* transient receptor potential cation channel, subfamily M, member 7	−1.84 ± 0.06	+1.73 ± 0.09
	*Monodelphis domestica* heat shock protein 84b	+5.51 ± 0.03	−1.82 ± 0.00
	*Mus musculus* WAPL protein	−1.83 ± 0.07	+2.14 ± 0.08
	*Oryzias javanicus* alpha-2-macroglobulin	+2.03 ± 0.03	−1.93 ± 0.03
	*Oryzias javanicus* complement C3-S	+1.93 ± 0.04	−1.94 ± 0.01
	*Oryzias javanicus* complement factor H-related protein	+1.94 ± 0.02	−2.36 ± 0.05
	*Oryzias javanicus* estrogen receptor alpha	+1.65 ± 0.09	−1.95 ± 0.05
	*Oryzias javanicus* protein kinase C and casein kinase substrate in neurons 2	+2.15 ± 0.02	−1.86 ± 0.03
	*Oryzias javanicus* translocase of inner mitochondrial membrane 13 homolog	+1.75 ± 0.06	−1.86 ± 0.06
	*Oryzias latipes* hox gene cluster	+1.65 ± 0.06	−1.67 ± 0.01
	*Oryzias latipes* translationally-controlled tumor protein	−1.72 ± 0.04	+1.83 ± 0.05
	*Solea senegalensis* glutathione *S* transferase Rho-class	+6.94 ± 0.06	−1.84 ± 0.09
Metabolism	*Danio rerio* importin 7 (ipo7), mRNA	+1.73 ± 0.07	−1.84 ± 0.03
	*Danio rerio* phospholipase A2, group XIIB	−1.53 ± 0.08	+1.52 ± 0.07
	*Danio rerio* uridine phosphorylase 2	−3.72 ± 0.07	+2.91 ± 0.08
	*Oryzias javanicus* apolipoprotein A1 precursor	+1.85 ± 0.04	−1.92 ± 0.07
	*Oryzias javanicus* phosphatidylinositol transfer protein beta isoform	+1.91 ± 0.05	−1.82 ± 0.03
	*Oryzias javanicus* phytanoyl-CoA hydroxylase	−1.94 ± 0.05	+1.83 ± 0.03
	*Oryzias javanicus* potassium large conductance calcium-activated channel, subfamily M, alpha member 1	+1.95 ± 0.08	−2.34 ± 0.04
	*Pseudopleuronectes americanus* apolipoprotein A1 precursor	+2.02 ± 0.04	−1.82 ± 0.02
	*Salmo salar* acetyl-CoA acetyltransferase	+1.84 ± 0.06	−1.94 ± 0.08
	*Spisula solidissima* mRNA for nerve hemoglobin (nHb gene)	−1.91 ± 0.08	+2.11 ± 0.02
Poorly characterized	*Danio rerio* arno protein (ARF exchange factor)	+1.83 ± 0.08	−1.84 ± 0.01
	*Danio rerio* DNA-directed RNA polymerase II subunit RPB2	−1.82 ± 0.08	+1.94 ± 0.08
	*Mus musculus* BAC clone RP24-324J2 from chromosome 10	−1.84 ± 0.04	+1.96 ± 0.07
	*Mus musculus* clone RP23-105K11	+1.92 ± 0.08	−1.83 ± 0.04
	*Oryzias javanicus* alanine-glyoxylate aminotransferase	+1.81 ± 0.02	−1.92 ± 0.02
	*Oryzias javanicus* TAR DNA binding protein	+1.82 ± 0.09	−2.01 ± 0.04
	*Oryzias javanicus* type II iodothyronine deiodinase	+1.73 ± 0.09	−1.91 ± 0.04
	*Oryzias melastigma* telomerase reverse transcriptase	−4.31 ± 0.01	+1.85 ± 0.05
	*Paralichthys olivaceus* heparin cofactor II	−1.81 ± 0.04	+2.44 ± 0.08
	*Sparus aurata* delta 6-desaturase	+1.92 ± 0.06	−6.21 ± 0.09
	*Tetraodon nigroviridis* full-length cDNA	−1.85 ± 0.04	+1.83 ± 0.08
	*Tetraodon nigroviridis* full-length cDNA	−1.84 ± 0.08	+2.12 ± 0.04
	*Tetraodon nigroviridis* full-length cDNA	+1.96 ± 0.08	−1.82 ± 0.07
	*Oryzias latipes* HN1-like protein	+1.92 ± 0.01	−2.01 ± 0.04

SW, seawater; FW, freshwater; +, upregulation; −, downregulation.

**Figure 1:**
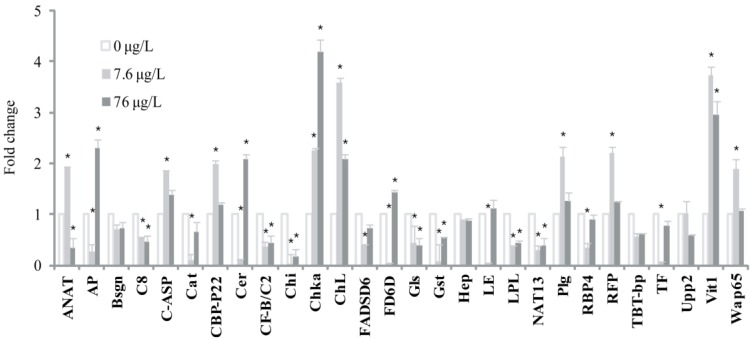
Expression changes of 28 selected genes in seawater *O. javanicu*s by bisphenol A exposure. Fish were exposed to 0, 7.6 and 76 μg/L bisphenol A in seawater for 48 h and RNAs were extracted from livers. The mRNA levels were evaluated by real-time quantitative RT-PCR and expressed relative to β-actin expression levels. Each histogram represents the mean ± SD (*n* = 3).

**Figure 2:**
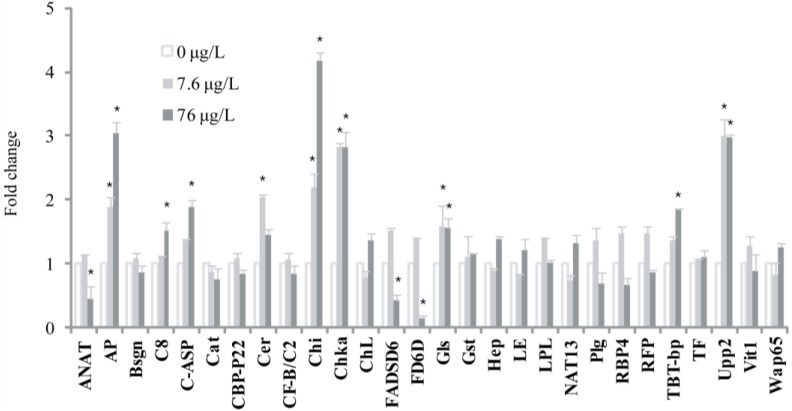
Expression changes of 28 selected genes in freshwater *O. javanicu*s by bisphenol A exposure. Fish were exposed to 0, 7.6 and 76 μg/L bisphenol A in freshwater for 48 h and RNAs were extracted from livers. The mRNA levels were evaluated by real-time quantitative RT-PCR and expressed relative to β-actin expression levels. Each histogram represents the mean ± SD (*n* = 3). * Significantly different from each 0 μg/L group (*p* < 0.05). Abbreviations: see Figure 1.

Among the 28 genes selected for the qRT-PCR experiment, 24 genes (excluding Bsgn, Hep, TBT-bp and Upp2) showed marked expression changed with an exposure to either 7.6 or 76 µg/L BPA in the seawater treatment (Figure 1). The genes for arylamine *N*-acetyl transferase, C1q-like adipose specific protein, calcium binding protein P22, choriogenin L, plasminogen, ring finger protein 141, vitellogenin 1, and warm temperature acclimation related 65 kDa protein were upregulated at the lower concentration of BPA (7.6 µg/L) but downregulated on exposure to 76 µg/L BPA. The expression of the choline kinase gene increased in a dose-dependent manner. In contrast, the apolipoprotein E1, catalase, ceruloplasmin, chitinase, delta-6 fatty acyl desaturase, delta 6-desaturase, glutathione S-transferase, leukocyte elastase inhibitor, lipoprotein lipase, *N*-acetyltransferase, retinol binding protein 4, and transferring genes were downregulated at the lower concentration of BPA, but upregulated at 76 µg/L BPA. However, expression of the chitinase, lipoprotein lipase, and *N*-acetyltransferase genes was strongly downregulated at both BPA concentrations. For fish exposed to BPA in FW, the microarray and qRT-PCR results were not significantly different (*p* < 0.05). The transcriptional changes of only 12 of these genes were statistically significant when fish were kept in FW and exposed to different concentration of BPA (Figure 2). For example, while in SW, mRNA expression of vittelogenin significantly increased in fish exposed to 7.6 µg/L BPA and decreased at 76 µg/L BPA, no significant change was observed in fish maintained in FW. The apolipoprotein E1, complement component C8 beta, C1q-like adipose specific protein, chitinase, choline kinase, and TBT-binding protein genes were upregulated in a dose-dependent manner, and the expression of the ceruloplasmin gene increased at the lower BPA concentration (7.6 µg/L) but decreased at 76 µg/L BPA. Expression of the arylamine *N*-acetyl transferase, delta-6 fatty acyl desaturase, and delta 6-desaturase genes did not change on exposure to 7.6 µg/L BPA, but decreased significantly at 76 µg/L BPA relative to the non-exposed control group (*p* < 0.05).

* Significantly different from each 0 μg/L group (*p* < 0.05). ANAT: Arylamine *N*-acetyl transferase; AP: Apolipoprotein E1; Bsgn: Basigin; C8: Complement component C8 beta; C-ASP: C1q-like adipose specific protein; Cat: Catalase; CBP-P22: Calcium binding protein P22; Cer: Ceruloplasmin; CF-B/C2: Complement factor B/C2-B; Chi: Chitinase; Chka: Choline kinase; ChL: Choriogenin L; FADSD6: Delta-6 fatty acyl desaturase; FD6D: Delta 6-desaturase; Gls: Glutaminase; Gst: Glutathione S-transferase; Hep: Hepcidin; LE: Leukocyte elastase inhibitor; LPL: Lipoprotein lipase; NAT13: *N*-acetyltransferase; Plg: Plasminogen; RBP4: Retinol binding protein 4; RFP: Ring finger protein 141; TBT-bp: TBT-binding protein; TF: Transferrin; Upp2: Uridine phosphorylase 2; Vit1: Vitellogenin 1; Wap65: Warm temperature acclimation related 65 kDa protein.

## 3. Materials and Methods

### 3.1. Animals, Exposure to BPA, and RNA Preparation

Male Javanese medaka (18–24 months old; 0.5–0.6 g; 3.5–3.7 cm) have been hatched in our laboratory for more than two years in freshwater. Two conditions have been used for these experiments: Freshwater (FW—salinity 0) and Seawater (SW—Salinity 33–34 at 24–25 °C). FW was prepared by mixing 8 mL of chloride/heavy metal neutralizer (Kukjefritz, Junjoo, Korea) into 40 L tap water, and aerating and aerated the mixture for two days in the laboratory at 24–25 °C. SW was pumped from the sea in front of our laboratory (KIOST, Geoje, Korea), filtered and used directly in our experiments. In each SW and FW condition, three groups of five fish were exposed to BPA, and three groups were included as non-exposed controls. The fish were transferred to 3 L beakers containing 2 L of SW or FW. Following acclimation for 48 h without food (to negate the effects of food consumption), the treatment groups were exposed to 76 µg/L BPA (Sigma-Aldrich, St. Louis, MO, USA) for 48 h. The BPA exposure concentration for the microarray experiment was based on the LC_50_ of BPA for *O. latipes* (*i.e.*, 760 µg/L, see [35]). To investigate the effect of a low BPA concentration on gene expression, we then used a 1/100 dilution (7.6 µg/L) of the LC_50_ concentration. The non-exposed controls were maintained in natural SW or FW.

At the end of each exposure experiment the liver of each fish was excised, and the total RNA was extracted independently from each group. For the quantitative real-time reverse-transcription (qRT-PCR) experiment, the fish were exposed to 76 or 7.6 µg/L BPA in SW for 48 h, the liver of each fish was excised and the RNA was extracted. TRIzol Reagent (Invitrogen, Carlsbad, CA, USA) was used for RNA purification, according to the manufacturer’s instructions. The pooled RNAs from the five fish in each group were used for the experiments.

### 3.2. Development of cDNA Libraries

*O. javanicus* cDNA libraries (standard, life stage-specific, heavy metal-specific) have been developed using the suppression subtractive hybridization technique. In brief, 20 adults and 20 juveniles were collected, and liver tissues pooled and mixed. Total RNA was extracted using an RNA extraction kit (Ambion, Austin, TX, USA) according to the manufacturer’s protocol. After DNase treatment the RNA concentration was determined using a NanoDrop ND-1000 spectrophotometer, and the quality was checked using a denaturing formaldehyde–agarose gel. We used the PCR Select cDNA Subtraction Kit (Clontech, Palo Alto, CA, USA) in combination with the Smart PCR cDNA Synthesis Kit (Clontech, Palo Alto, CA, USA) for construction of the library by suppression subtractive hybridization. The clones were ligated using a TA-vector system (pGEM-T Easy Vector System, Promega, Madison, WI, USA) and transformed by heat shock. Clones were amplified using vector-specific primers (M13 forward primer, M13 reverse primer), and the PCR products were sequenced using an ABI Prism 3100 Genetic Analyzer (Applied Biosystems, Foster City, CA, USA). Following sequencing, the cDNA fragments were identified based on their similarity to sequences in the National Center for Biotechnology Information (NCBI, Bethesda, MD, USA) database determined using the Basic Local Alignment Search Tool (BLAST). The sequences were compared with DNA and protein databases using blastn and tblastx analyses, respectively.

### 3.3. Preparation of cDNA Probes and Microarray Hybridization

Sequence-verified cDNA clones containing a set of 2500 genes from an *O. javanicus* library (Ecotoxicogenomics Laboratory, Korea Institute of Ocean Science and Technology, Geoje, Korea) were used to construct an *O. javanicus* cDNA microarray (OjaArray), which was printed by GenomicTree Inc. (Daejon, Korea). The cDNA clones were amplified by PCR and the PCR products were purified using the Qiagen PCR Purification Kit (Qiagen, Hilden, Germany). The purified cDNA clones were fabricated on aminosilane-coated microarray slides (UltraGAPS™, Corning, MA, USA) using a robotic microarrayer (GeneMachine, OmniGrid II, Genomic Instrumentation Services Inc., San Carlos, CA, USA). Each 50 µg of total RNA was reverse transcribed in the presence of Cy3- or Cy5-dUTP (NEN Life Sciences, Boston, MA, USA) at 42 °C for 2 h. The control RNA was labeled with fluorescent Cy3-dUTP and the test RNA was labeled with fluorescent Cy5-dUTP. Both the Cy3- and Cy5-labeled cDNAs were purified using a PCR purification kit (Qiagen, Hilden, Germany), following the manufacturer’s instructions. The purified cDNA was resuspended in 100 µL of hybridization solution containing 5× saline sodium citrate (SSC), 0.1% SDS, 30% formamide, 20 µg of human Cot-1 DNA, 20 µg of polyA RNA, and 20 µg of yeast tRNA (Invitrogen, Carlsbad, CA, USA). The hybridization mixtures were heated to 100 °C for 2–3 min, then pipetted directly onto the microarrays. The arrays were hybridized at 42 °C for 12–16 h in a humidified hybridization chamber (GenomicTree Inc., Daejeon, Korea). The hybridized microarrays were washed twice with 2 × SSC/0.1% SDS for 5 min, 0.1 × SSC/0.1% SDS for 10 min, and 0.1 × SSC for 2 min. The washed microarrays were immediately dried in a microarray centrifuge (GenomicTree Inc., Daejeon, Korea).

### 3.4. Data Acquisition and Identification of Differentially Expressed Genes

The hybridization images were analyzed using GenePix Pro 4.0 software (Axon Instruments, Union City, CA, USA). The average fluorescence intensity of each spot was calculated and the local background was subtracted. All data normalization and the selection of those genes showing changes in expression were performed using GeneSpring 7.1 software (Silicon Genetics, Redwood City, CA, USA). Reliable identification of genes was based on the cut-off value of the two-component error model [36], following intensity-dependent normalization (lowess). The averages of the normalized ratios were calculated by dividing the mean normalized signal channel intensity by the mean normalized control channel intensity. Student *t*-tests were performed to identify those genes that were differentially expressed in the exposed and non-exposed fish, based on a significance level of *p* < 0.05.

### 3.5. Gene Ontology Clustering

Classification of the genes that were upregulated or downregulated following treatment (SW + BPA and FW + BPA) or in the controls (SW or FW only) was undertaken using GeneSpring 7.1 software according to clusters of orthologous groups of proteins.

### 3.6. Quantitative Real-Time Reverse-Transcription PCR and Statistical Analysis

To evaluate and confirm the observations from the microarray hybridization, we performed quantitative real-time reverse-transcription PCR (qRT-PCR) using 28 randomly selected sets of primers for various functional groups (the primers used in the qRT-PCR are shown in Supplementary Table S2). The thermal condition for PCR included 40 cycles at 95 °C for 30 s, 60 °C for 30 s and 72 °C for 30 s. The transcriptional changes in 28 genes (arylamine *N*-acetyl transferase, apolipoprotein E1, basigin, complement component C8 beta, C1q-like adipose specific protein, catalase, calcium binding protein P22, ceruloplasmin, complement factor B/C2-B, chitinase, choline kinase, choriogenin L, delta-6 fatty acyl desaturase, delta 6-desaturase, glutathione *S*-transferase, hepcidin, leukocyte elastase inhibitor, lipoprotein lipase, *N*-acetyltransferase, plasminogen, retinol binding protein 4, ring finger protein 141, TBT-binding protein, transferrin, uridine phosphorylase 2, vitellogenin 1, and warm temperature acclimation-related 65 kDa protein) in fish exposed to 7.6 and 76 µg/L BPA were quantified using qRT-PCR, as described previously [37]. The quantitative PCR analyses were performed independently three times. The β-actin gene of Javanese medaka (*actb*; GenBank accession no. DQ660327) was used as an internal control following confirmation of stable transcription from the BPA exposure microarray data [38]. The group means were compared using ANOVA, followed by Duncan’s test for multiple comparisons. A value of *p* < 0.05 indicated statistical significance.

## 4. Conclusions

The above findings need to be considered in terms of the possible roles of osmoregulatory mechanisms in chemical uptake and toxicity, as has been shown in many reports of metal exposure [18,39], in which it has also been suggested that changes in salinity may alter the toxicity of certain chemicals as a consequence of molecular-level responses in exposed organisms. Consequently, environmental stressors such as salinity can potentially affect the toxicity of environmental pollutants (including BPA) in the euryhaline fish *O. javanicus*, through regulation of the activation or inactivation of transcription of responsive genes. BPA exposure revealed the occurrence of a number of DEGs, and salinity-associated induction of various functional groups of genes in *O. javanicus*. This study suggests that transcriptional changes induced by environmental pollutants might be regulated differently in single species under environmental salinity. It also suggests that the identification of specific biomarkers using microarrays, and experiments conducted under a wider range of exposure conditions could improve the accuracy of chemical risk assessments. As *O. javanicus* is highly adaptable to environmental salinity, it is a potential model animal for investigating the toxic effects of chemicals in both estuarine and marine environments.

## Supplementary Files

Supplementary File 1 Supplementary Information (PDF, 108 KB)
