# Supplementary material for: Transcriptional Changes Caused by Bisphenol A in Oryzias javanicus, a Fish Species Highly Adaptable to Environmental Salinity
Source: Mar Drugs. 2014 Feb 14;12(2):983–98. doi: 10.3390/md12020983 (PMC3944526; doi:10.3390/md12020983)
Supplement: Supplementary File 1 — Supplementary Information (PDF, 108 KB) [file marinedrugs-12-00983-s001.pdf]

Supplementary Information

Table S1. The list of differentially expressed genes by BPA exposure and comparative expression values in SW and FW (FC > 3).

Best hit description	SW	FW
Gallus gallus proline rich protein 6 (PRR6), mRNA	−5.99 ±0.01	+1.01 ±0.06
Oryzias javanicus si:dkey-183c16.7	−4.98 ±0.04	+1.18 ±0.02
Canis familiaris ribosomal protein S14, transcript variant 4	+3.11 ±0.02	−1.12 ±0.05
Carassius auratus protein phosphatase 2A regulatory subunit B delta isoform mRNA	+3.02 ±0.01	−1.21 ±0.01
Chymotrypsinogen 2-like protein, mRNA	+2.22 ±0.05	+3.00 ±0.01
Danio rerio choline kinase alpha (chka), mRNA	−1.42 ±0.05	+3.01 ±0.06
Danio rerio mitochondrial fission regulator 1, nuclear gene encoding mitochondrial protein	+3.12 ±0.06	−1.28 ±0.08
Danio rerio protein tyrosine phosphatase-like (proline instead of catalytic arginine), member b	−3.23 ±0.01	−1.10 ±0.01
Danio rerio similar to Fatty acid synthase, mRNA	−1.02 ±0.07	−3.00 ±0.09
Danio rerio uridine phosphorylase 2, mRNA	−3.77 ±0.11	+2.98 ±0.08
Danio rerio vertebrate Yip1 interacting factor homolog B	−4.09 ±0.03	+1.10 ±0.04
Dicentrarchus labrax sex hormone-binding globulin mRNA	+3.01 ±0.14	−1.30 ±0.20
Fundulus heteroclitus TBT-binding protein, mRNA	+4.24 ±0.03	+1.84 ±0.04
Gasterosteus aculeatus clone CNB112-D01 mRNA sequence	+3.10 ±0.09	−1.27 ±0.01
Homo sapiens ATP citrate lyase (ACLY), transcript variant 2, mRNA	+3.02 ±0.08	+1.57 ±0.04
Macaca mulatta similar to TRAF-binding protein	−3.17 ±0.04	−1.02 ±0.05
Monodelphis domestica similar to heat shock protein 84b	+5.53 ±0.15	−1.50 ±0.14
Oncorhynchus mykiss chitinase	+1.11 ±0.15	+4.17 ±0.11
Oreochromis niloticus decorin mRNA	+2.97 ±0.14	+6.70 ±0.23
Oryzias dancena retinoid X receptor beta	−3.42 ±0.14	−1.10 ±0.22
Oryzias hubbsi proteasome subunit, beta type 8	−1.31 ±0.13	+3.53 ±0.21
Oryzias javanicus apolipoprotein E1	−1.20 ±0.06	+3.05 ±0.23
Oryzias javanicus hematopoietic SH2 domain containing	−3.45 ±0.08	−1.05 ±0.07
Oryzias javanicus HSPC038 protein	−3.05 ±0.06	−1.17 ±0.02
Oryzias javanicus warm-temperature-acclimation-related-65 kDa-protein	+5.88 ±0.07	+1.40 ±0.03
Oryzias latipes cyclin B2 (ccnb2), mRNA	+3.02 ±0.06	−1.18 ±0.04
Oryzias latipes DNA, MHC class I region	−4.18 ±0.06	+1.12 ±0.04
Oryzias latipes vitellogenin II	−3.69 ±0.06	−1.01 ±0.08
Oryzias melastigma telomerase reverse transcriptase	−4.31 ±0.07	+1.65 ±0.22
Osmerus mordax glycerol-3-phosphate dehydrogenase, cytoplasmic	−3.03 ±0.19	−1.84 ±0.01
Salmo salar translation initiation factor eIF-2B subunit epsilon	+3.01 ±0.27	+1.44 ±0.23
Salmo salar transmembrane protein 85	+5.22 ±0.11	−1.26 ±0.21
Solea senegalensis glutathione S transferase	+6.90 ±0.01	−1.59 ±0.12
Sparus aurata putative delta 6-desaturase (FD6D) mRNA	+1.96 ±0.42	−6.29 ±0.13
Takifugu rubripes apolipoprotein A-IV4	+2.94 ±0.21	+3.99 ±0.34
Tetraodon nigroviridis full-length cDNA	−4.27 ±0.17	−1.08 ±0.18
Tetraodon nigroviridis full-length cDNA	+3.88 ±0.12	+1.51 ±0.21
Tetraodon nigroviridis full-length cDNA	−1.72 ±0.01	+3.01 ±0.11
Transducer of ERBB2 (TOB1)	+3.02 ±0.21	+1.30 ±0.01

SW, seawater; FW, freshwater; +, upregulation; −, downregulation.

Table S2. Primers list of real-time quantitative RT-PCR.

Primers	Genes		Sequences
ANAT	Arylamine *N*-acetyl transferase	F	5′-CATTCACTGTGGTGAGAGGA-3′
		R	5′-TCCAAAGCTCACGTCTGTAA-3′
AP	Apolipoprotein E1	F	5′-GGATCCATACTTCTCTCAGGT-3′
		R	5′-GTCTGAACATGTTTCCATGAG-3′
Bsgn	Basigin	F	5′-ATCCTCGTGACCATCATCTT-3′
		R	5′-AGGAACTTTGCAGAAAGCAC-3′
C8	Complement component C8 beta	F	5′-GTGCACTATAACCCCGACTT-3′
		R	5′-AGTGACCTCACAGCTCCTTC-3′
C-ASP	C1q-like adipose specific protein	F	5′-CTGTTGACGTTGTTGCTTTG-3′
		R	5′-AAAAACTACTGGGGTTCGTG-3′
Cat	Catalase	F	5′-GCGGTACAACAGCGCCGATGA-3′
		R	5′-GGATGGACGGCCTTCAAGTTCT-3′
CBP-P22	Calcium binding protein P22	F	5′-CCACAGTCAGATCACTCGAC-3′
		R	5′-CAAAGTGCAGCTTGTTCATC-3′
Cer	Ceruloplasmin	F	5′-GTCAGTTTGAAGTGGTCTCTG-3′
		R	5′-GAACCTATGAATCCTCCTTGT-3′
CF-B/C2	Complement factor B/C2-B	F	5′-CGTGGACATATGTGACCCTA-3′
		R	5′-TGTCCAAATCTGTTGGAATG-3′
Chi	Chitinase	F	5′-GATGCAGGATAGAGAGAGGTT-3′
		R	5′-AGATCCATGGAAGTCATAGGT-3′
Chka	Choline kinase	F	5′-CCAGAAGTACCCCTCAAAGA-3′
		R	5′-TTCTTCTGCTGGAAGTAGGC-3′
ChL	Choriogenin L	F	5′-TGCAACACCAGGAAACTATG-3′
		R	5′-TTTTCGGCTCATACCAATAC-3′
FADSD6	Delta-6 fatty acyl desaturase	F	5′-TGGACAATGACTTTCCTGTG-3′
		R	5′-CCTGAAGATATTGGGTTTG-3′
FD6D	Delta 6-desaturase	F	5′-TCTCGTGTCATTAGCCACTAT-3′
		R	5′-ATTCTTCTCTGCCTGAACTCT-3′
Gls	Glutaminase	F	5′-TGAGTCAGGAAGTGTCATGG-3′
		R	5′-TTACCAAGGCGATCTAAAGG-3′
Gst	Glutathione *S*-transferase	F	5′-TCGACAAGATGGAGCACAAATC-3′
		R	5′-CAGTTTGTTCCCCTGAGACTTGA-3′
Hep	Hepcidin	F	5′-AACTGCTGCAAGAACTACAAG-3′
		R	5′-CCAACAGCCTTTATCTGTTT-3′
LE	Leukocyte elastase inhibitor	F	5′-GTTTGATGCAAAGGAAACTC-3′
		R	5′-CATGAAGTTCTCGTAGGTCAG-3′
LPL	Lipoprotein lipase	F	5′-TTCACTGTTTGGAACTCACG-3′
		R	5′-ACCAGATCAGCAAACTCTCC-3′
NAT13	*N*-acetyltransferase	F	5′-TCGAGATCATCGAGACAAAA-3′
		R	5′-GCAGAAATTGCCTTTGAAAC-3′
Plg	Plasminogen	F	5′-TGCTGAGCAGATCTCAACAT-3′
		R	5′-AATGTAAGCGAAGGATTTGC-3′
RBP4	Retinol binding protein 4	F	5′-AGAACCAGGTATGCAGGTGT-3′
		R	5′-ACCCCAGTATTTCATCCTGA-3′

Table S2. *Cont.*

RFP	Ring finger protein 141	F	5′-TCTGCACCAAGATCAACAAG-3′
		R	5′-TTTGTCGATGCATTTCTGAC-3′
TBT-bp	TBT-binding protein	F	5′-CCATCAATGTTAGCCACAAA-3′
		R	5′-CGTAATGTATTCGGGTTCCT-3′
TF	Transferrin	F	5′-TCACACAGACTCCTTCCTCTA-3′
		R	5′-TCAGTCACAGATCAGATCCTC-3′
Upp2	Uridine phosphorylase 2	F	5′-GACATTCTGTACCACTTCAGC-3′
		R	5′-CCACTTTGTACATGGAGTAGC-3′
Vit1	Vitellogenin 1	F	5′-ACAAAAGGTTCCACTCTCAGC-3′
		R	5′-CCAACTTTAACCTCCATCTCC-3′
Wap65	Warm temperature acclimation related 65 kDa protein	F	5′-CGACAAGGGTCAAACTTTTT-3′
		R	5′-TTTGGATAGCCTTCCTCAAG-3′

© 2014 by the authors; licensee MDPI, Basel, Switzerland. This article is an open access article distributed under the terms and conditions of the Creative Commons Attribution license (http://creativecommons.org/licenses/by/3.0/).
